# Supplementary material for: Empirical methods for controlling false positives and estimating confidence in ChIP-Seq peaks
Source: BMC Bioinformatics. 2008 Dec 5;9:523. doi: 10.1186/1471-2105-9-523 (PMC2628906; doi:10.1186/1471-2105-9-523)
Supplement: Additional File 1 — A variety of html documents from the USeq web site detailing the available applications, their best usage, output file type descriptions, command line menus, etc. [file 1471-2105-9-523-S1.zip › USeqUserGuides/JavaDoc.zip!/JavaDoc/bioroot/PatternMatch.html]

PatternMatch


|  |  |  |
| --- | --- | --- |
| **Overview** \| **Package** \| **Class** \| **Use** \| **Tree** \| **Deprecated** \| **Index** \| **Help** | |  |
| **PREV CLASS**   **NEXT CLASS** | **FRAMES**    **NO FRAMES**     **All Classes** |  |
| SUMMARY: NESTED \| FIELD \| CONSTR \| METHOD | DETAIL: FIELD \| CONSTR \| METHOD |  |


---


## bioroot Class PatternMatch

```
java.lang.Object
  bioroot.PatternMatch
```

---

``` public class PatternMatch extends java.lang.Object ```

Class to hold information and helper methods relating to pattern matches.

---

**Constructor Summary**

`PatternMatch(int start,
int end,
java.lang.String match)`


| **Method Summary** | |
| --- | --- |
| `static PatternMatch[]` | `arrayListToArray(java.util.ArrayList arrayList)`             Convert an ArrayList of PatternMatch to PatternMatch[] |
| `static PatternMatch[]` | `mapIt(java.util.regex.Matcher matcher)`             Returns an array of hits (PatternMatch[]) given a Matcher. |
| `static java.util.ArrayList` | `mapItArrayList(java.util.regex.Matcher matcher)`             Returns an ArrayList of PatternMatch given a Matcher. |
| `java.lang.String` | `toString()` |

**Methods inherited from class java.lang.Object**

`equals, getClass, hashCode, notify, notifyAll, wait, wait, wait`

**Constructor Detail**

### PatternMatch

```
public PatternMatch(int start,
                    int end,
                    java.lang.String match)
```


**Method Detail**

### toString

```
public java.lang.String toString()
```

:   **Overrides:**: `toString` in class `java.lang.Object`

---


### mapIt

```
public static PatternMatch[] mapIt(java.util.regex.Matcher matcher)
```

:   Returns an array of hits (PatternMatch[]) given a Matcher.

---


### mapItArrayList

```
public static java.util.ArrayList mapItArrayList(java.util.regex.Matcher matcher)
```

:   Returns an ArrayList of PatternMatch given a Matcher.

---


### arrayListToArray

```
public static PatternMatch[] arrayListToArray(java.util.ArrayList arrayList)
```

:   Convert an ArrayList of PatternMatch to PatternMatch[]


---


|  |  |  |
| --- | --- | --- |
| **Overview** \| **Package** \| **Class** \| **Use** \| **Tree** \| **Deprecated** \| **Index** \| **Help** | |  |
| **PREV CLASS**   **NEXT CLASS** | **FRAMES**    **NO FRAMES**     **All Classes** |  |
| SUMMARY: NESTED \| FIELD \| CONSTR \| METHOD | DETAIL: FIELD \| CONSTR \| METHOD |  |


---
